# Viruses in bronchiectasis: a pilot study to explore the presence of community acquired respiratory viruses in stable patients and during acute exacerbations

**DOI:** 10.1186/s12890-018-0636-2

**Published:** 2018-05-22

**Authors:** Alicia B. Mitchell, Bassel Mourad, Lachlan Buddle, Matthew J. Peters, Brian G. G. Oliver, Lucy C. Morgan

**Affiliations:** 1 Respiratory Cellular and Molecular Biology, Woolcock Institute of Medical Research, The University of Sydney, Sydney, NSW 2006 Australia; 2 Department of Respiratory Medicine, Concord Repatriation General Hospital, Concord, NSW 2139 Australia; 3 Concord Clinical School, University of Sydney, Sydney, NSW 2006 Australia; 4 Molecular Biosciences, School of Life Sciences, University of Technology Sydney, Building 4, 15 Broadway, Ultimo, NSW 2007 Australia; 5 Centre for Health Technologies, University of Technology Sydney, Sydney, NSW 2007 Australia; 6 Emphysema Centre, Woolcock Institute of Medical Research, The University of Sydney, Sydney, NSW 2006 Australia

**Keywords:** Bronchiectasis, Respiratory viruses, Viral infection, Influenza

## Abstract

**Background:**

Bronchiectasis is a chronic respiratory condition. Persistent bacterial colonisation in the stable state with increased and sometimes altered bacterial burden during exacerbations are accepted as key features in the pathophysiology. The extent to which respiratory viruses are present during stable periods and in exacerbations is less well understood.

**Methods:**

This study aimed to determine the incidence of respiratory viruses within a cohort of bronchiectasis patients with acute exacerbations at a teaching hospital and, separately, in a group of patients with stable bronchiectasis. In the group of stable patients, a panel of respiratory viruses were assayed for using real time quantitative PCR in respiratory secretions and exhaled breath. The Impact of virus detection on exacerbation rates and development of symptomatic infection was evaluated.

**Results:**

Routine hospital-based viral PCR testing was only requested in 28% of admissions for an exacerbation. In our cohort of stable bronchiectasis patients, viruses were detected in 92% of patients during the winter season, and 33% of patients during the summer season. In the 2-month follow up period, 2 of 27 patients presented with an exacerbation.

**Conclusions:**

This pilot study demonstrated that respiratory viruses are commonly detected in patients with stable bronchiectasis. They are frequently detected during asymptomatic viral periods, and multiple viruses are often present concurrently.

## Background

Bronchiectasis is a progressive disease characterised by permanent dilatation of bronchi, impairment of mucociliary clearance, and retention of secretions. Recurrent respiratory infections are a key feature of bronchiectasis, with the majority of research focusing on the role of bacteria in stable patients, during acute exacerbations and particularly in disease progression [1, 2]. Despite significant advances in diagnostic immunology and radiology, and a growing global awareness of bronchiectasis as a significant twenty-first century clinical problem, the underlying cause of bronchiectasis in a given patient is not always clear. Approximately 40% of cases remain idiopathic [3], after the most common causes (immunodeficiencies, cystic fibrosis (CF), primary ciliary dysfunction (PCD), allergic bronchopulmonary aspergillosis (ABPA), connective tissue disorders, chronic obstructive pulmonary disease (COPD)-related, or asthma-related) have been excluded [4, 5].

Respiratory infections in early childhood are an important cause of airway damage with the potential to initiate the vicious cycle of epithelial damage, airway dilatation, mucostasis, and bacterial colonisation [6]. Prior to widespread vaccination in the mid twentieth century, measles and pertussis played a major role in post-infectious damage leading to bronchiectasis [7]. The incidence and mortality of pneumonia associated with influenza and pneumococcal infection has also been reduced in both paediatric [8–10] and adult populations with access to vaccination programs [11, 12]. Pneumonia in childhood caused by common respiratory viruses has been associated with significant early airway damage and these viruses are emerging as major factors in the subsequent development of bronchiectasis [4, 13]. While research has focused on defining the aetiology of bronchiectasis due to its implications in individualised treatment and management of the disease, little work has been done to define the role of respiratory viruses in stable and acute bronchiectasis.

As there is considerable phenotypic overlap between bronchiectasis, CF and COPD, the basic understanding gained from investigating the role of viruses in exacerbations and asymptomatic viral detection during stable phases in these diseases may guide our knowledge regarding bronchiectasis.

The association between viral infection and bacterial superinfection is well described in the literature, and more recently, with changes in the microbiome. In COPD and CF, respiratory viruses precipate exacerbations, which in turn, are associated with accelerated disease progression [14]. Mallia et al. [15] demonstrated that experimental rhinovirus infection in patients with COPD could induce symptoms associated with exacerbations, and induce changes in the microbiota. These findings in COPD have been further confirmed by a serial analysis of the lung microbiome following rhinovirus infection [16]. In patients with CF, significantly higher levels of respiratory viruses were detected during exacerbations (46%) compared to stable phases (17%) [17]. The detection of viruses during exacerbation has also been associated with an increase in colony counts of Pseudomonas aeruginosa, suggesting that viruses may also affect the stability of the microbiome in cystic fibrosis [18]. In these diseases, increased viral presence was often observed during exacerbations which also lead to changes in the resident microbial communities. Bacterial colonisation is a common and key feature of the pathophysiology of bronchiectasis. Less is known about the role of viruses in stable state bronchiectasis, or the effect of viruses on the equilibrium between symbiotic and pathogenic bacterial species.

Therefore, this pilot study aimed to determine the incidence of respiratory virus testing ordered by physicians within a cohort of bronchiectasis patients with acute exacerbations at a teaching hospital and separately, to determine the incidence of viral detection within a group of patients with stable bronchiectasis to establish baseline viral prevalence. The incidence of symptomatic viral infections and rates of exacerbations in this cohort was also evaluated.

## Methods

### Part 1

A retrospective clinical audit was undertaken to determine the rate of testing for respiratory viruses for patients admitted to Concord Repatriation General Hospital July 2011 to June 2016 with an acute exacerbation of bronchiectasis. Patient data regarding exacerbation frequency, previous lung function and hospital admissions were collected from the Australian Bronchiectasis Registry.

### Part 2

#### Clinical measures

Two cohorts of patients were recruited from an outpatient clinic whilst clinically stable. All patients attending the specialised bronchiectasis clinic during the recruitment months were asked to participate. Bronchiectasis was deemed to be clinically stable from the point of view by the consultant physician in clinic based on the patient’s history, and no deterioration in clinical symptoms in the month prior to their clinic visit. A history of viral-related symptoms was not an exclusion criteria. One cohort was recruited during the winter months in Australia (May – September), while the other was recruited during the summer months (January – March). Samples were collected from each patient during their clinic visit, to determine if viruses were present within the lungs of bronchiectasis patients when clinically stable, similar to resident bacterial species. This is a tertiary referral centre for PCD, where the diagnosis of PCD was made based on ciliary motility studies and electron microscopy. Patients provided a basic medical history and filled out a common cold questionnaire at the time of recruitment [19]. The common cold questionnaire (CCQ) assesses viral symptoms on an 11-point scale. Based on the presence or absence of these symptoms, the questionnaire predicts the likelihood of a viral infection. Results are classified into three categories; ‘no virus’, ‘possible virus’ or ‘probable virus’ depending on how many symptoms are reported [19]. The results of the questionnaires were considered at the time of analysis in conjunction with the viral PCR results, and were not used as inclusion or exclusion criteria.

Spirometry was performed at the time of sample collection (according to ATS/ERS guidelines) [20] and compared to previous results to ensure that patients were at baseline. FEV_1_ was used as a surrogate measure of severity in this cohort of patients. The filters from the spirometer mouthpieces were frozen during storage, then processed for RNA extraction from exhaled breath using a methodology described previously [21], and spontaneously expectorated sputum samples were also collected. All patients were reviewed by the physiotherapist in clinic if sputum was not easily spontaneously expectorated.

To investigate if asymptomatic infections could develop into acute exacerbations, information regarding exacerbations and hospitalisations in the following 2 months were collected for all patients. Other patient outcomes, including lung function, acute viral or bacterial infections, were also collected.

#### Sample molecular processing

Filters and sputum samples were analysed for a panel of respiratory viruses using PCR. Virus RNA was extracted from the exhaled breath captured in spirometry filters using a methodology published previously [21]. Filters were first removed from the spirometry mouthpieces and 1 ml of Bioline Lysis Buffer RLY (Bioline, Alexandria, Australia) was then added. This was centrifuged for 2 min at 10 000 rpm. The eluate was collected after the final spin and stored at − 20 °C until RNA extraction. Sputum samples were homogenised by mixing the secretion with 1 ml of 1% B-ME Lysis Buffer RLY to achieve a final volume of 1.5 ml, which was then stored at − 20 °C. Following this, RNA was purified using the Isolate II RNA Mini Kit (Bioline, Alexandria, Australia) before conversion to cDNA using the Bioline SensiFAST cDNA Synthesis Kit (Bioline, Alexandria, Australia).

cDNA was assayed by uniplex real time reverse transcription polymerase chain reactions for human rhinovirus (HRV), respiratory syncytial virus (RSV), influenza virus type A and influenza virus type B, parainfluenza virus (PIV) 1, 2 and 3, and human metapneumovirus (HMPV). Real Time quantitative PCR (qPCR) assays utilised the StepOnePlus Real-Time PCR System (Applied Biosystems, ThermoFisher, Massachusetts, USA). All samples were run in triplicate, with 2 μl of cDNA template added to Bioline SensiFAST Probe Hi-ROX Master Mix. PCR primers were sourced from the literature [21–25], and have been previously optimised using clinical samples. Forward and reverse primers were added along with virus specific probe. The qPCR was run for 40 cycles, and the cycle threshold (CT) value was defined for each reaction.

#### Statistical analysis

T-tests were used to compare parametric data sets, Mann-Whitney tests for non-parametric data, and Fisher’s exact test was completed for contingency table analyses using GraphPad Prism version 6.

## Results

### Part 1

During the study period 47 patients were identified from the Bronchiectasis Registry as having been admitted to Concord Repatriation General Hospital for an exacerbation of bronchiectasis with a total of 83 admissions. The average age for this cohort was 72 ± 14 years, mean ± SD (range 24–88) (male = 19).

Of the 83 total admissions, viral PCR was requested in only 23. In comparison, bacterial and fungal cultures were requested in 73 admissions.

Viral PCR was positive in 9 of 23 cases (39%), with 3 cases of influenza A and 6 cases of HRV.

Bacterial and fungal cultures were positive in 22/73 admissions (30%). The most commonly detected pathogen by culture was *Pseudomonas aeruginosa* in 9 admissions, followed by *Haemophilus influenzae* in 7 cases, *Burkholderia cepacia* in 1, and *Achromobacter xylosoxidans* in 1 case. Fungal species were less common, with *Aspergillus* spp. detected during 3 exacerbations, and *Candida albicans* in 1 case.

### Part 2

#### Winter cohort

Twelve patients with stable bronchiectasis were recruited in the winter cohort. The clinical characteristics of these patients are summarised in Table 1. Four patients were on maintenance therapy with an inhaled corticosteroid (ICS) /long acting beta agonist (LABA) combination inhaler, while the majority had been prescribed a short acting beta agonist (SABA) as needed. Only one patient reported being a past smoker, all other patients had never smoked.

**Table 1:** Summary of patient characteristics and respiratory virus detection and exacerbation rates in both the winter and summer cohorts

Season	Mean age ± SD	Gender (F/M)	Mean FEV_1_ ± SD	Comorbid PCD	Overall viral detection rate	Number of patients with Influenza	Multiple virus detection rate	Exacerbation rate during follow up period
Winter (*n* = 12)	36 ± 12	10/2	77% ± 22%	9/12 (75%)	11/12 (92%)	10/12 (83%)	10/12 (83%)	0/12 (0%)
Summer (*n* = 15)	60 ± 17	12/3	60% ± 33%	3/15 (20%)	5/15 (33%)	5/15 (33%)	0/15 (0%)	1/15 (7%)
Overall (*n* = 27)	49 ± 19	22/5	67% ± 29%	12/27 (44%)	16/27 (59%)	15/27 (56%)	10/27 (37%)	1/27 (4%)

Of the 12 patients with bronchiectasis recruited during the winter period, 9 of these also had a concurrent diagnosis of PCD. The majority of these patients (11/12) had relatively preserved lung function with an FEV_1_ greater than the lower limit of normal. One patient had severely reduced lung function, with an FEV_1_ of only 21% predicted (0.56 L), and an FEV_1_/FVC ratio of 50% based on ATS/ERS guidelines [26].

All patients completed the CCQ on the day of secretion sampling. None reported enough symptoms on the 11-point scale to be categorised as “probable virus”. All patients remained stable, with no reported exacerbations or hospital admissions within a month prior to or 2 months after sample collection.

Filters and sputum samples were processed for a panel of respiratory viruses. Nine of 12 patients had respiratory virus RNA identified in filter samples. In the filters, influenza was the most commonly detected respiratory virus (9/12), with 3 patients having influenza A, 3 with influenza B, 2 with concurrent influenza A and B detection and one patient who demonstrated co-detection of human rhinovirus and influenza A (Table 2). Using qPCR, in the samples where the same virus was detected in the filter as the sputum sample, the CT value was lower (approximately 33 cycles) compared with those viruses found in the sputum alone (approximately 37 cycles).

**Table 2:** Specific viruses detected in the filter and sputum samples of patients in Summer and Winter cohorts

Patient	Winter	
	Filter positive	Sputum positive
1	Flu A	RV, RSV, Flu A + B
2	Flu B	RV, RSV, Flu A + B
3	Flu B	RV, RSV, Flu A + B
4	Flu B	RV, RSV, Flu A + B
5	Flu A	RV, RSV + Flu A
6		RV + RSV
7		Flu A
8	RV + Flu A	RV + Flu A
9	Flu A + B	RV, RSV, Flu A + B
10	Flu A + B	RV, RSV, Flu A + B
11	Flu A	RV, RSV, Flu A + B
12		
Patient	Summer	
	Filter positive	Sputum positive
13		
14	FluA	FluA
15		
16		
17		
18	FluA	FluA
19		FluA
20		FluA
21		
22		
23		
24		
25		
26	FluA	FluA
27		

In sputum samples, 11 of 12 patients had a respiratory virus identified. A single patient had only influenza A identified. Co-infection was more common with 7 patients showing concurrent detection of HRV, RSV, influenza A and B; 1 patient with HRV, RSV and influenza A; 1 with HRV and RSV; 1 with HRV and influenza A. All 9 subjects with virus detected in exhaled breath also had virus detected in the matched sputum sample. As 11 of 12 patients in the winter cohort were viral positive, it was not possible to correlate virus detection with disease severity based on FEV1. Similarly, a correlation between viral detection and use of SABA, or ICS/LABA combination treatment could not be deduced.

#### Summer cohort

Fifteen patients were recruited in the summer cohort. Their clinical characteristics are summarised in Table 1. Ten patients were on maintenance therapy with an ICS/LABA combination inhaler, two had additional tiotropium therapy; 12/15 had been prescribed a SABA PRN. All patients reported never smoking.

In this cohort, 3 of 15 patients had a concomitant PCD diagnosis, while 2 of 15 also had a diagnosis of asthma. This was a slightly more severe cohort of bronchiectasis patients based on spirometry when compared with the group recruited during the winter season. The mean FEV_1_ in this group was 59% of predicted, however this was not significantly different to the winter group. Only one patient reported symptoms of viral infection at the time of sample collection, the rest of the patients reported feeling well at the time of their clinic visit which was confirmed by responses to the common cold questionnaire. Two patients with severe bronchiectasis (one with comorbid asthma) were subsequently admitted to hospital for an exacerbation within 2 months of sample collection.

During the summer season, respiratory viruses were less commonly detected, with 3 of 15 patients demonstrating influenza A detection in the filters and 5 of 15 samples detecting Influenza A in the sputum sample (Table 2). No other respiratory viruses on our panel were detected in these samples. Furthermore, none of the patients in the summer cohort had a “probable virus” based on the CCQ. In the patients who were viral positive in the summer cohort, the average FEV1 was lower (*p* > 0.05), compared with the viral negative group. However, no associations between medication usage and viral detection were observed.

One patient was admitted to hospital within 2 weeks of the clinic visit with an exacerbation of bronchiectasis, and influenza A was again detected in both exhaled breath and sputum samples. Another patient who experienced an exacerbation 6 weeks after their clinic visit, did not have any viruses detected in either sample.

#### Comparison of cohorts

**Fig. 1:**
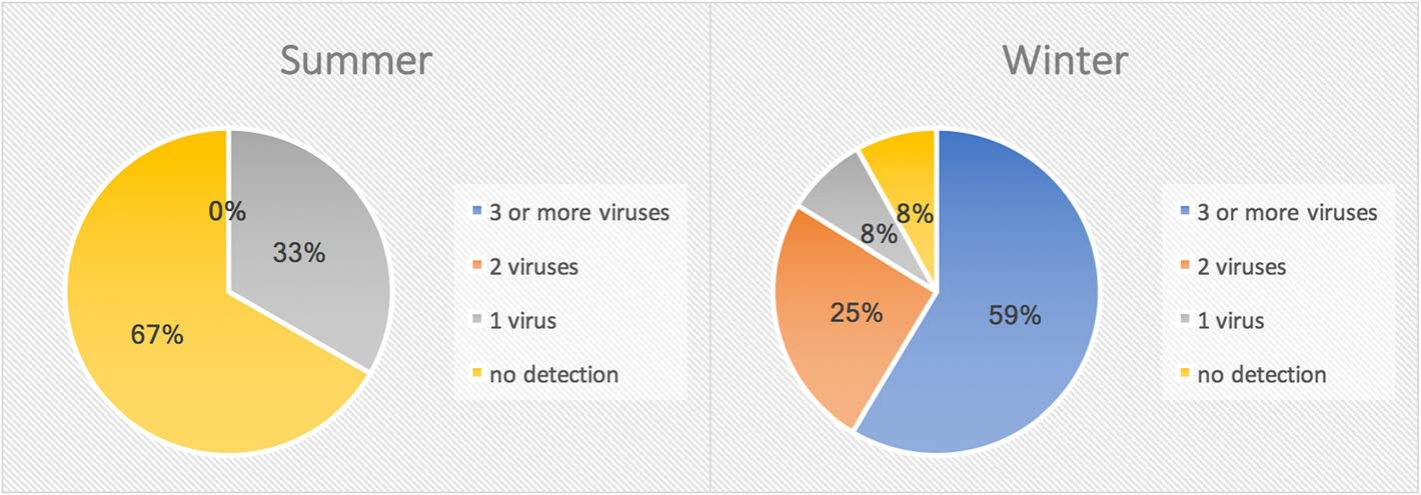
Rates of single or multiple respiratory virus detection during stable state in the summer and winter cohorts

There was a significant difference in viral detection between Summer and Winter cohorts (*p* < 0.01), with a greater rate of viral detection observed during the winter months (Fig. 1). These cohorts were not age or severity matched, and there was a significantly higher rate of underlying PCD in the winter cohort (*p* < 0.05).

## Discussion

Our small retrospective audit of admissions for exacerbation of bronchiectasis revealed how infrequently viral PCR testing was requested in a large teaching hospital with ready access to on site rapid respiratory viral PCR. Reflecting the current state of the literature, bacterial and fungal species were more frequently assumed to be the etiological agents and therefore, tested for in the majority of patients presenting with an exacerbation. Viral PCR testing was only requested in 28% of the bronchiectasis exacerbations included in the audit, compared with 88% of admissions where bacterial and fungal culture were requested.

However, despite the greater frequency of request for bacterial and fungal culture, viruses were still detected in 39% of the samples when viral PCR was requested, compared with bacterial or fungal pathogen detection in 30% of samples sent for testing. It is important that sampling rates increase, and prospective, longitudinal studies of both bacterial and viral pathogens in stable and exacerbating bronchiectasis are undertaken, if we are to understand with more precision, the role of viruses in exacerbations and their seasonality.

Due to the low rate of viral testing in acute exacerbations of bronchiectasis at our centre, we designed a study to determine the incidence of respiratory virus detection during stable periods and whether this was associated with an increased risk of exacerbation or developing a symptomatic viral infection. Studying stable patients with bronchiectasis provides information regarding the background level of viruses to inform future analysis of results obtained during acute exacerbations. Our pilot study demonstrated that respiratory viruses are commonly detected in respiratory secretions and the exhaled breath of patients with stable bronchiectasis. They are frequently detected during asymptomatic periods, and multiple viruses are often present concurrently.

In this study, there was a 92% detection rate in the winter cohort, and a 33% detection rate in the summer cohort. Other studies have detection rates of around 20% in stable bronchiectasis rising to around 40–50% in exacerbations in non-CF bronchiectasis in adults [27, 28]. One potential reason for obtaining such high virus detection by PCR is contamination within the PCR reaction. We are confident that high viral detection rates in the winter cohort this is not caused by poor PCR technique or experimental contamination as out negative controls were always negative. Furthermore, whilst the samples were collected during different periods of the year, the PCRs were carried out simultaneously. However, we used a highly sensitive PCR which can detect as few as 5 virions. In our study, even low CT values were classified as viral positive whereas in other studies these might be classified as negative. Whist not a part of this study, we have compared our research lab virus PCR results to virus positivity by PCR obtained from a diagnostic lab. We found almost 100% agreement for all viruses, apart from rhinovirus, where we found our PCR was more sensitive (twice the detection frequency). We think that the most plausible explanation for the high sample detection in our winter cohort happened to be sampled during a year that was recognised to have a heavy burden of influenza infections. Other possible reasons might be the increased severity of bronchiectasis based on FEV_1_ values in our cohort, and also, the high incidence of PCD as our clinic is a state-wide referral centre for PCD. However, it is unusual to have found such high rates of multiple virus detection, and further studies are needed in PCD to confirm these findings. There is currently little literature regarding the detection and persistence of respiratory viruses in the respiratory tract of individuals with PCD.

Respiratory viruses were more common during winter season, compared with the summer season. This study confirms previously reported seasonality of respiratory viruses [29] for RSV, however rhinovirus has been shown to occur all year round in respiratory specimens which was not observed in this cross-sectional study of bronchiectasis patients. Influenza has also demonstrated peak detection during the winter months in temperate zones, and year-round distribution in tropical areas [30]. In this study, we observed a heavy burden of influenza during the winter season, however influenza A virus was still detected in multiple asymptomatic individuals during the summer months.

Detection of respiratory viruses in the exhaled breath and sputum samples of this bronchiectasis cohort, was not significantly associated with disease severity or risk of exacerbation within the 2 month follow up period. In the one patient who was admitted to hospital with a bronchiectasis exacerbation within 2 weeks of their clinic visit, influenza A was present within both the exhaled breath and sputum sample. However, this was the only case where an exacerbation was associated with virus detection in our study. The short duration of this follow up time may not be adequate, however, to make a clear determination of exacerbation risk in this cohort. A longitudinal study design with regular viral sampling during periods of both stable disease and exacerbation, and more in depth analysis of patient outcomes may be needed to elucidate this risk.

No association was observed between viral detection and treatment with ICS/LABA or SABA alone. In the summer cohort, viruses were more commonly detected in patients with more severe disease as indicated by spirometry, with 80% patients who had influenza A detection demonstrating an FEV_1_ below 30% predicted based on the GLI-2012 reference set [31]. In the winter cohort, viral detection had no significant association with spirometry values.

Real time PCR allowed quantification of viral load, with higher viral load detection in the sputum sample predicting detection in the exhaled breath sample collected using the spirometry filters. Huang et al. [32] showed that presence of influenza virus within the respiratory tract is necessary but not sufficient to cause a symptomatic influenza infection. Host immune responses play an important role, and activation of multiple simultaneous pattern recognition receptors to cause antiviral and inflammatory responses are associated with symptomatic infection. Individuals who retain tight control over these responses usually remain asymptomatic, and may explain why asymptomatic infection was so prevalent in our cohort. These patients with bronchiectasis all have chronic bacterial lung colonisation, which may play a role in downregulating immune responses [33].

A surprising finding was that of influenza A detection only during the summer months. Traditionally, influenza A activity peaks during the winter months and viruses such as rhinovirus are more commonly seen in summer and early autumn. The Australian influenza surveillance network showed that there was a higher than normal level of influenza A detected during January to March of 2017, the sampling period of our summer cohort. Likely due to the fact that this group of bronchiectasis patients have an underlying respiratory disease, and impaired muco-ciliary clearance, it is possible that these individuals were more susceptible to acquiring these circulating viruses.

A large proportion of those recruited during the winter months had PCD as the underlying cause of bronchiectasis, as this study was undertaken at a tertiary referral centre for PCD. Ciliary dysmotility impairs mucociliary clearance and it seems plausible that this might result in persistence of viral nucleic acids within sputum, even if the virus is not actively replicating. Whilst the number of subjects in this pilot is small, it raises the possibility that differences in underlying pathophysiology of bronchiectasis extend to heterogeneity in the pathogenesis of viruses.

Since the introduction of culture-independent techniques, a substantial increase in bacterial detection has been observed [34]. Molecular methods that identify bacterial species based on nucleic acid presence has greatly improved diagnostic accuracy [35, 36], and has allowed discovery of a whole range of bacterial species that are present within the lower respiratory tract. The introduction of these molecular based methods such as PCR, have also allowed the detection of respiratory viral species to become faster and easier [37]. This greatly increased the rate of respiratory infections that were found to be attributable to viruses, as this is a much more sensitive and specific tool. This was an important step in realising the high frequency of respiratory viral infections, and thus their importance in clinical disease. It also allowed a more guided approach to treatment, with a decrease in the use of antibacterial agents in some cases. Characterising the role of viruses in both stable bronchiectasis and during exacerbations may allow a greater understanding of disease pathogenesis.

## Conclusions

Our pilot study provides preliminary data supporting the notion that respiratory viruses are an important part of the lung microbiome in patients with bronchiectasis. The high rates of respiratory virus detection in patients with stable bronchiectasis encourages further studies in this area to determine how viruses may impact both chronic and transient bacterial species within the lung, and the role that viruses may have in exacerbations. This is the first study to investigate the potential impact of viruses in bronchiectasis. Many fundamental questions have been raised regarding the role of respiratory viruses in this disease process, and as outlined, recent advances in metagenomic techniques have provided the tools to investigate this area. We are just beginning to understand the role of viruses in many chronic respiratory diseases and it is now timely to apply this work in patients with bronchiectasis.

## Abbreviations

ABPA: Allergic bronchopulmonary aspergillosis
ATS: American thoracic society
CCQ: Common cold questionnaire
CF: Cystic fibrosis
COPD: Chronic obstructive pulmonary disease
CT: Cycle threshold
ERS: European respiratory society
HMPV: Human metapneumovirus
HRV: Human rhinovirus
PCD: Primary ciliary dyskinesia
PCR: Polymerase chain reaction
PIV: Parainfluenza virus
qPCR: Quantitative polymerase chain reaction
RSV: Respiratory syncytial virus
